# Mendelian randomization analyses explore the relationship between inflammatory bowel disease and genitourinary diseases

**DOI:** 10.1097/MD.0000000000048759

**Published:** 2026-05-15

**Authors:** Hongliang Cao, Chengdong Shi, Xianyu Dai, Bo Yuan, Gang Yang, Song Wang

**Affiliations:** a Department of Urology, The First Hospital of Jilin University, Changchun, Jilin, China.

**Keywords:** Crohn disease, genitourinary diseases, inflammatory bowel disease, Mendelian randomization study, ulcerative colitis

## Abstract

Inflammatory bowel disease (IBD), such as Crohn disease (CD) and ulcerative colitis, plays a significant role in the development of various conditions, including genitourinary diseases (GDs). Nevertheless, the precise relationship between IBD and GDs must still be more adequately understood. This research utilized a Mendelian randomization (MR) approach to identify the underlying relationships between IBD and 27 GDs. Data from the Integrative Epidemiology Unit Open genome-wide association studies project summarized findings from genome-wide association studies. Five MR analyses were used to investigate the impact of IBD on GDs, primarily utilizing the inverse-variance weighted (IVW) method for causal effects assessment. Moreover, sensitivity analyses were utilized to examine the heterogeneity and pleiotropy of the results. The IVW method revealed results suggesting that CD may be associated with an elevated risk of hypertensive renal disease (IVW odds ratio (OR) = 1.17, 95% confidence interval (CI): 1.05–1.31, *P* value = .0036), diabetic nephropathy (IVW OR = 1.05, 95% CI: 1.00–1.11, *P* value = .049), chronic kidney disease (IVW OR = 1.06, 95% CI: 1.02–1.11, *P* value = .0069), chronic renal failure (IVW OR = 1.04, 95% CI: 1.01–1.06, *P* value = .0034), calculus of kidney and ureter (IVW OR = 1.07, 95% CI: 1.03–1.11, *P* value = .00070), neuromuscular dysfunction of bladder (IVW OR = 1.08, 95% CI: 1.02–1.16, *P* value = .014), and hydronephrosis (IVW OR = 1.07, 95% CI: 1.01–1.14, *P* value = .027). Moreover, ulcerative colitis was potentially linked to an increased likelihood of neuromuscular dysfunction of bladder (IVW OR = 1.10, 95% CI: 1.00–1.21, *P* value = .046) and prostatitis (IVW OR = 1.12, 95% CI: 1.04–1.20, *P* value = .0019). Results obtained from MR-Egger, weighted median, simple mode, and weighted mode methods were similar to those of the IVW approach. Moreover, heterogeneity and pleiotropy are unlikely to distort according to the sensitivity analysis results. In conclusion, our findings indicate a potential link between IBD and certain GDs; however, additional confirmation is required.

## 1. Introduction

Inflammatory bowel disease (IBD) is a chronic gastrointestinal disorder caused by the immune system, with Crohn disease (CD) and ulcerative colitis (UC) being the main types. Globally, the incidence is increasing, with a rising burden worldwide. Due to its complex pathogenesis and costly clinical management, it poses a significant challenge globally.^[1]^ IBD impacts the digestive system and affects various parts of the body, with extraintestinal manifestations often seen in CD and UC. These extraintestinal manifestations often affect the musculoskeletal system, skin, liver and biliary tract, and eyes, which can significantly impact patients’ survival and quality of life, making their management vital to intervening in IBD.^[2,3]^ Furthermore, the development of IBD is influenced by an intricate combination of genetic and environmental factors that impact the function of the intestinal barrier, immune system, and microbial imbalance.^[4]^

Recent research, both epidemiological and experimental, indicates that individuals with IBD may have a higher likelihood of developing conditions affecting the genitourinary system. However, it is still unclear whether specific mechanisms exist between them. A study conducted in Denmark from 1977 to 2018 with 75,236 patients diagnosed with IBD and 767,403 controls found that individuals with IBD were twice as likely to develop urolithiasis after being diagnosed with IBD and had a 42% higher risk before the diagnosis.^[5]^ A comprehensive analysis of data from multiple centers showed a clear link between IBD, specifically CD, and a higher likelihood of developing kidney cancer. Moreover, individuals with IBD in Asian nations face a notably higher likelihood of developing prostate cancer, particularly those with UC.^[6]^ An analysis using propensity scores to match patients in the US nationwide inpatient sample from 2016 to 2018 suggests that individuals with CD have a higher likelihood of experiencing nephrolithiasis, tubulointerstitial nephritis, any stage of chronic kidney disease (CKD), and moderate-to-severe CKD when compared to those without IBD.^[7]^ A review highlighted the connection between IBD and kidney and urinary system issues, such as nephritis, kidney stones, bladder fistulas, urinary tract cancer, medication-induced kidney damage, glomerulonephritis, tubulointerstitial disease, and renal amyloidosis, indicating a significant relationship between IBD and urogenital conditions.^[8]^

Mendelian randomization (MR) analysis is a valuable method in epidemiology that investigates the causal relationship between 2 characteristics by utilizing genetic variants as instrumental variables (IVs). This can help reduce the influence of potential confounding factors and decrease the chance of reverse causation.^[9]^ In this study, we conducted a 2-sample MR analysis to explore the relationship between IBD and the risk of developing 27 genitourinary diseases (GDs).

## 2. Materials and methods

### 2.1. Study design

**Figure 1.**
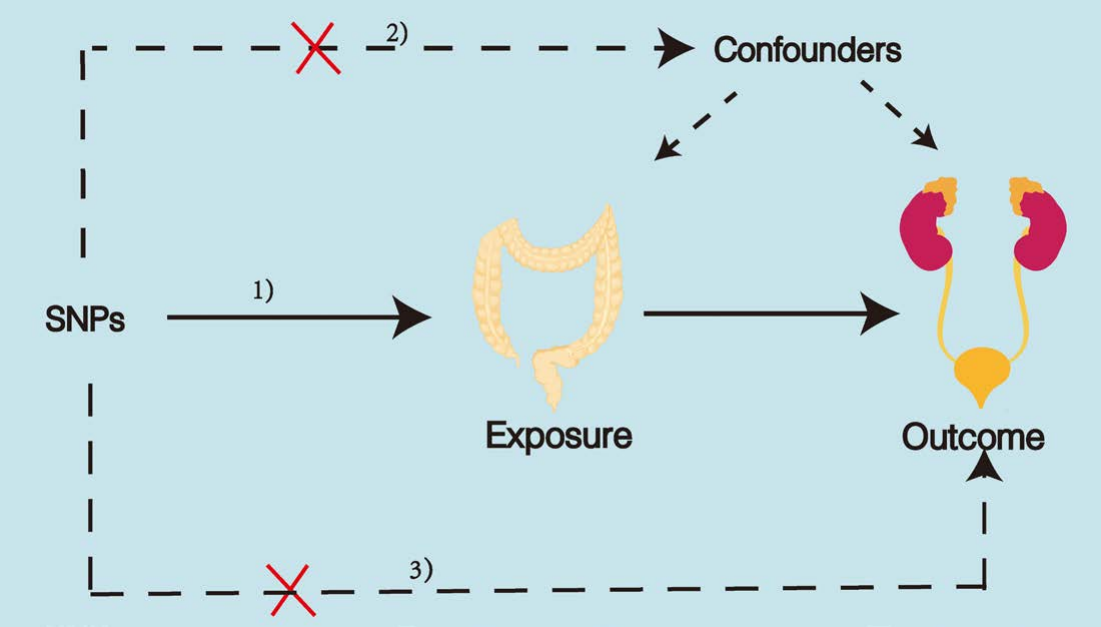
Three key assumptions of MR analysis. CD = Crohn disease, GDs = genitourinary diseases, IVs = instrumental variables, MR = Mendelian randomization, UC = ulcerative colitis.

Using genome-wide association studies (GWAS) summary data for IBD and GDs, this study identified suitable IVs for MR analysis to investigate the causal link between IBD and GDs. This research adhered closely to the 3 assumptions of MR analysis: the IVs chosen were linked to the exposure, the IVs were not associated with any potential confounding factors, the IVs can influence outcomes solely through the exposure (see Fig. 1). The datasets utilized in this research are accessible to the public. The original studies had obtained ethical approval and written consent from participants.

### 2.2. GWAS statistics source

Data summaries from GWAS for IBD and GDs were acquired from the Integrative Epidemiology Unit OpenGWAS project (website: https://gwas.mrcieu.ac.uk/), and the data on IBD has been used by previous article.^[10]^ This is a large website where GWAS data can be accessed online for free. Table 1 displays the specifics of the GWAS data utilized in this article.

**Table 1:** The detailed information on GWAS used in this MR analysis.

Trait	GWAS ID	Sample size	Consortium	nSNPs
Crohn disease	ebi-a-GCST004132	40,266	NA	9,457,998
Ulcerative colitis	ebi-a-GCST004133	46,975	NA	9,474,559
Malignant neoplasm of kidney	finn-b-C3_KIDNEY_NOTRENALPELVIS	218,792	FinnGen_r5	16,380,466
Hypertensive Renal Disease	finn-b-I9_HYPTENSRD	163,305	FinnGen_r5	16,380,163
Diabetic nephropathy	finn-b-DM_NEPHROPATHY_EXMORE	184,987	FinnGen_r5	16,380,336
Chronic tubulointerstitial nephritis	finn-b-N14_CHRONTUBULOINTNEPHRITIS	201,648	FinnGen_r5	16,380,412
Membranous nephropathy	ebi-a-GCST010005	7979	NA	5,327,688
IgA nephropathy	ebi-a-GCST90018866	477,784	NA	24,182,646
Chronic nephritic syndrome	finn-b-N14_CHRONEPHROPATH	215,404	FinnGen_r5	16,380,439
Cystic kidney disease	finn-b-Q17_CYSTIC_KIDNEY_DISEA	218,448	FinnGen_r5	16,380,465
Chronic kidney disease	finn-b-N14_CHRONKIDNEYDIS	216,743	FinnGen_r5	16,380,459
Chronic renal failure	ebi-a-GCST90018822	482,858	NA	24,185,976
Calculus of kidney and ureter	finn-b-N14_CALCUKIDUR	218,414	FinnGen_r5	16,380,464
Bladder cancer	finn-b-C3_BLADDER	218,792	FinnGen_r5	16,380.466
Cystitis	finn-b-N14_CYSTITIS	203,221	FinnGen_r5	16,380,426
Neuromuscular dysfunction of bladder	finn-b-N14_NEUROMUSCDYSBLADD	196,427	FinnGen_r5	16,380,413
Calculus of lower urinary tract	finn-b-N14_CALCULOWER	214,067	FinnGen_r5	16,380,457
Prostate cancer	ieu-b-85	140,254	PRACTICAL	20,346,368
Benign prostatic hyperplasia	finn-b-N14_PROSTHYPERPLA	85,917	FinnGen_r5	16,378,414
Prostatitis	finn-b-N14_PROSTATITIS	74,658	FinnGen_r5	16,377,460
Stress incontinence	ukb-b-9873	463,010	MRC-IEU	9,851,867
Urethral stricture	finn-b-N14_URETHRALSTRICT	159,927	FinnGen_r5	16,380,411
Malignant neoplasm of testis	finn-b-C3_TESTIS	95,213	FinnGen_r5	16,378,835
Hydronephrosis	finn-b-N14_HYDRONEPHR	202,482	FinnGen_r5	16,380,414
Testicular hypofunction	finn-b-E4_TESTICHYPO	93,150	FinnGen_r5	16,378,751
Male infertility	finn-b-N14_MALEINFERT	73,479	FinnGen_r5	16,377,329
Female infertility	finn-b-N14_FEMALEINFERT	75,450	FinnGen_r5	16,377,038
Sexual dysfunction	finn-b-F5_SEXDYS	213,986	FinnGen_r5	16,380,450
Erectile dysfunction	finn-b-ERECTILE_DYSFUNCTION	95,178	FinnGen_r5	16,378,833

GWAS = genome-wide association studies, IEU = Integrative Epidemiology Unit, MR = Mendelian randomization, NA = not applicable, nSNPs = number of single nucleotide polymorphisms.

### 2.3. Selection of IVs

We selected single nucleotide polymorphisms (SNPs) highly associated with IBDs and with significant genome-wide significance (*P* value < 5 × 10^–8^) as potential IVs. Following this, we eliminate linkage disequilibrium and establish the threshold as *R*^2^ < 0.001, kb = 10,000. We removed palindromic variants for incompatible alleles. The selected SNPs’ strength was evaluated by computing the *F*-statistic using the given equation:


$$\begin{array}{l} F=\frac{R^{2}\left(N-2\right)}{\left(1-R^{2}\right)} \end{array}$$

*R*^2^ represents the proportion of variance in exposure that can be accounted for by the IVs, N is the sample size. SNPs were removed if the *F*-statistic was less significant than 10. We then removed outcome-related SNPs (*P* value < 5 × 10^–5^). We conducted the Steiger test before performing each MR analysis to avoid reverse causality, and SNPs with TRUE results were included. The MR-Pleiotropy RESidual Sum and Outlier (PRESSO) examination was performed to remove any outliers in the dataset.^[11]^ In addition, due to the intricate interplay between genetics and environmental factors that contribute to IBDs and GDs, it is challenging to discern confounding elements that impact both. Therefore, we did not consider potential confounding factors in our analysis, in line with approaches previously documented in literature.^[12–17]^ The selected SNPs were the last IVs in the subsequent MR analyses.

### 2.4. MR analysis and sensitivity analysis

**Figure 2.**
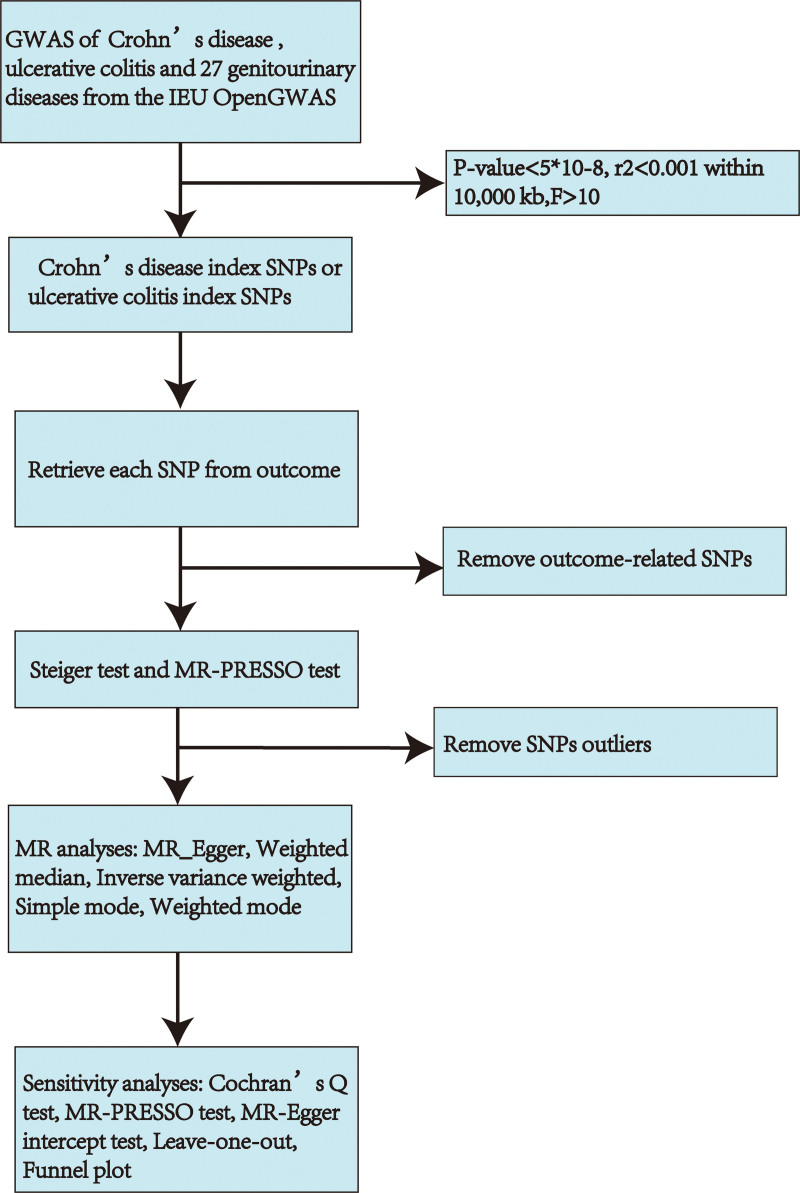
Workflow chart of MR discovering the casual association between IBD and 27 GDs. GDs = genitourinary diseases, GWAS = genome-wide association study, IBD = inflammatory bowel diseases, MR = Mendelian randomization, SNP = single nucleotide polymorphisms,.

Five MR methods, including MR-Egger, weighted median, inverse-variance weighted (IVW), simple mode, and weighted mode, were utilized in the MR analysis.^[10]^ The IVW estimates were chosen as the primary approach, with 4 additional methods used to enhance their reliability.^[18]^ A statistical significance was determined with a significance level below 0.05. The heterogeneity of MR results was evaluated using Cochran *Q* test, which utilized IVW and MR-Egger methods.^[19]^ If the *P* value was below .05, it was deemed heterogeneous, leading to using a random-effect model for further analysis. Moreover, the MR-Egger regression and MR-PRESSO methods were employed to evaluate and rectify pleiotropy. The procedures included evaluating horizontal pleiotropy, correcting for outliers, and determining if the causal effect changed significantly after removing outliers (*P* < .05 means horizontal pleiotropy).^[11,20]^ Furthermore, a leave-one-out analysis was performed to evaluate if an individual SNP influenced or skewed the MR estimate. A funnel plot was implemented to assess the likely pleiotropy. Figure 2 shows the workflow of this MR. Statistical analyses were conducted with R software version 4.3.1, using the TwoSampleMR R package (v0.5.7) and MR-PRESSO (v1.0).

## 3. Results

**Figure 3.**
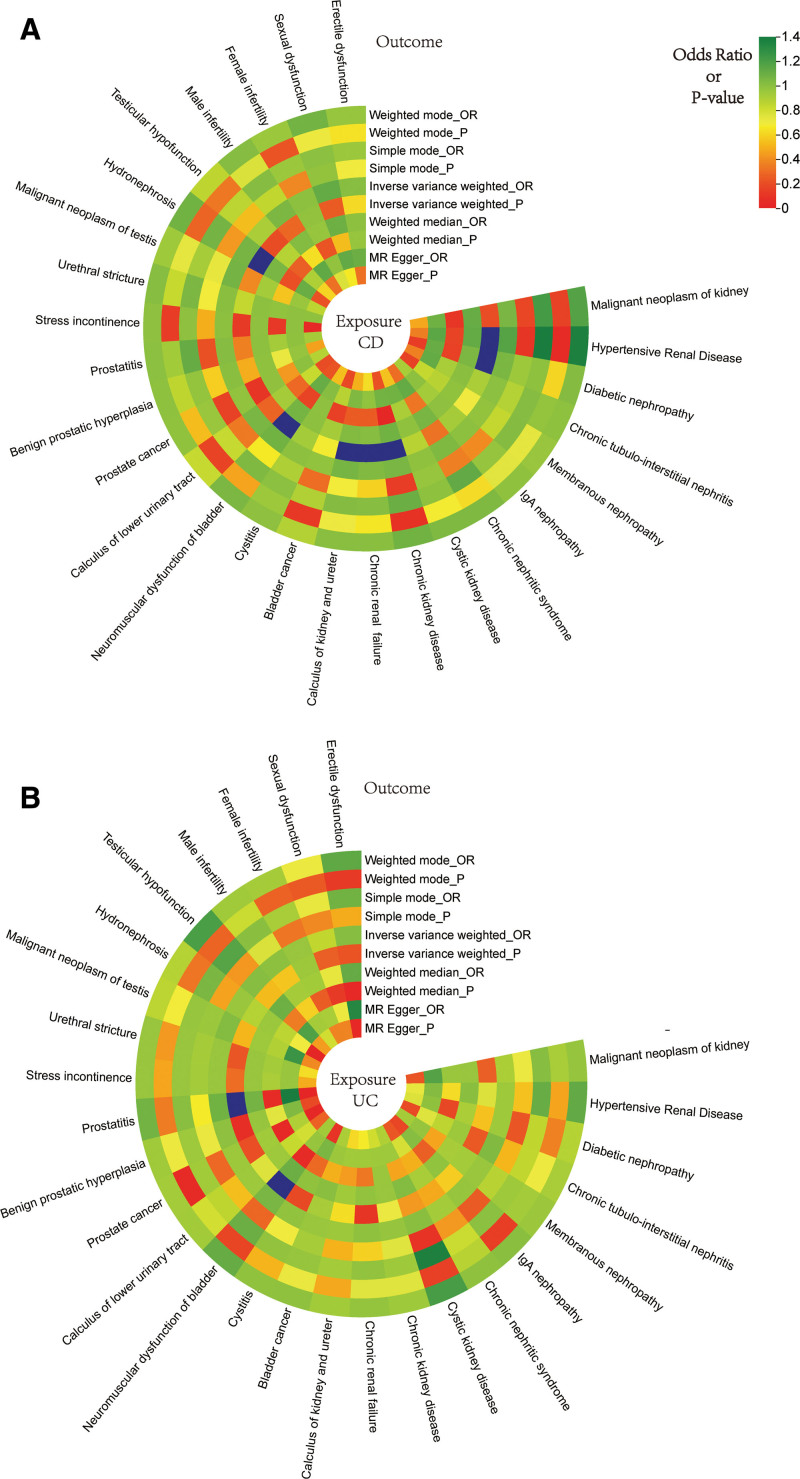
(A) Ring heat map illustrates the MR analysis results showing the impact of CD on GDs; (B) Ring heat map illustrates the MR analysis results showing the impact of UC on GDs. CD = Crohn disease, GDs = genitourinary diseases, MR = Mendelian randomization, UC = ulcerative colitis.

**Figure 4.**
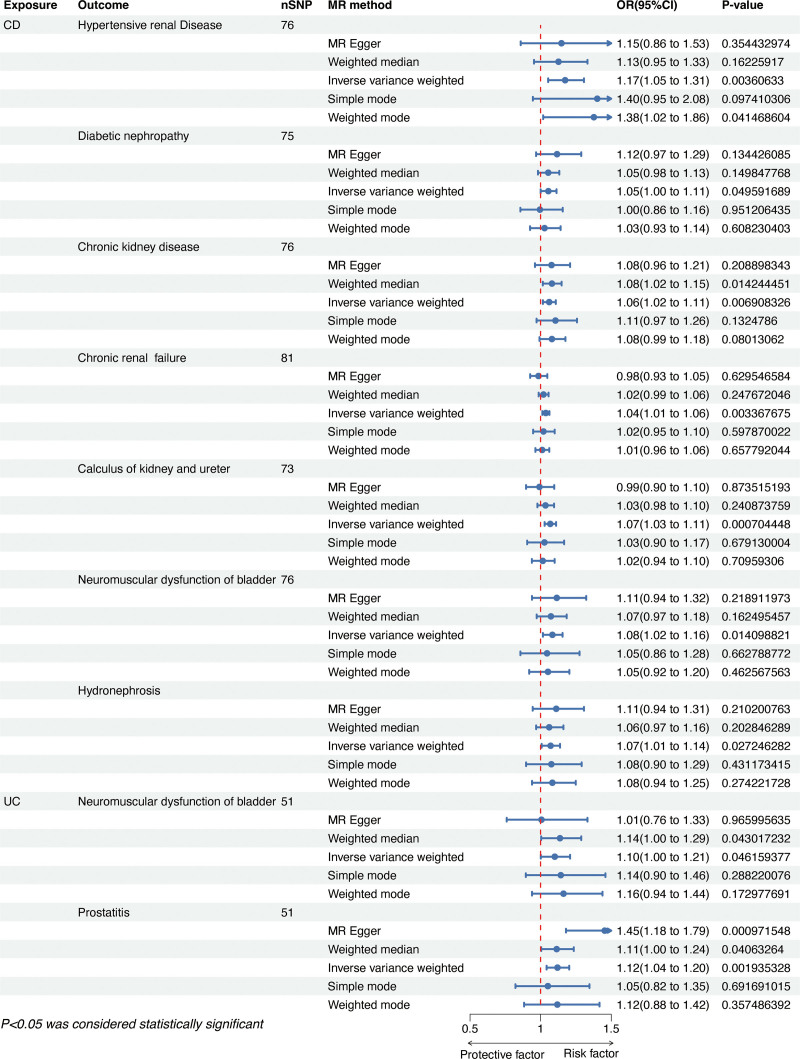
The positive results of MR analysis by using the IVW method. CD = Crohn disease, GDs = genitourinary diseases, IVW = inverse-variance weighted, MR = Mendelian randomization, UC = ulcerative colitis.

We screened SNPs under the process described in Figure 2; after removing the outliers suggested by the MR-PRESSO method, we finally got the IVs for the following MR analysis (Table S1,S2,S3,S4,S5,S6,S7,S8 and S9). The rough results of the 2-sample MR analysis between IBD and GDs are displayed in Figure 3, with dark blue squares indicating *P* values below .05 using the IVW method. As shown in Figure 4, the outcomes of the IVW method revealed that CD was potentially linked to an higher risk of hypertensive renal disease (IVW odds ratio [OR] = 1.17, 95% confidence interval [CI]: 1.05–1.31, *P* value = .0036), diabetic nephropathy (IVW OR = 1.05, 95% CI: 1.00–1.11, *P* value = .049), CKD (IVW OR = 1.06, 95% CI: 1.02–1.11, *P* value = .0069), chronic renal failure (IVW OR = 1.04, 95% CI: 1.01–1.06, *P* value = .0034), calculus of kidney and ureter (IVW OR = 1.07, 95% CI: 1.03–1.11, *P* value = .00070), neuromuscular dysfunction of bladder (IVW OR = 1.08, 95% CI: 1.02–1.16, *P* value = .014), and hydronephrosis (IVW OR = 1.07, 95% CI: 1.01–1.14, *P* value = .027). Additionally, UC was potentially linked to increased chances of neuromuscular dysfunction of bladder (IVW OR = 1.10, 95% CI: 1.00–1.21, *P* value = .046) and prostatitis (IVW OR = 1.12, 95% CI: 1.04–1.20, *P* value = .0019). The scatter plot of positive results is shown in Figure S1,S2,S3,S4,S5,S6,S7,S8 and S9 (A). The outcomes obtained from MR-Egger, weighted median, simple mode, and weighted mode techniques were similar to those of the IVW method (Fig. 4).

For sensitivity analysis, we identified heterogeneity between CD and Diabetic nephropathy (*P* < .05) using the IVW and MR-Egger techniques of Cochran *Q* test (Table 2). Thus, a random-effect model of IVW method was applied for subsequent analysis. Additionally, the MR-PRESSO technique identified horizontal pleiotropy (*P* < .05), and even after eliminating outliers, the heterogeneity persisted (*P* < .05) (Table 2). Additionally, MR-Egger regression test revealed directional pleiotropy between UC and prostatitis (Table 2). Furthermore, no heterogeneity was found through Cochran *Q* test (*P* > .05) or pleiotropy through MR-PRESSO and MR-Egger regression methods (*P* > .05) (Table 2). In the Leave-one-out sensitivity analysis (Fig. S1,S2,S3,S4,S5,S6,S7,S8 and S9 (B)), no individual SNP significantly undermined the overall impact of IBD on GDs. The symmetrical funnel plot also suggests the absence of pleiotropy (Fig. 1,2,3,4,5,6,7,8 and 9 (C)).

**Table 2:** The sensitivity analysis results of MR with positive results using IVW method.

Exposure	Outcome	*P* value from IVW of Cochran *Q* test	*P* value from MR-Egger of Cochran *Q* test	*P* value from Intercept of MR-Egger test	*P* value from MR-PRESSO global outlier test
CD	Hypertensive renal disease	.432	.400	.871	.426
CD	Diabetic nephropathy	.0006	.0006	.403	.001
CD	Chronic kidney disease	.060	.052	.778	.065
CD	Chronic renal failure	.210	.264	.081	.250
CD	Calculus of kidney and ureter	.165	.199	.123	.146
CD	Neuromuscular dysfunction of bladder	.516	.487	.729	.531
CD	Hydronephrosis	.451	.426	.635	.438
UC	Neuromuscular dysfunction of bladder	.070	.064	.509	.061
UC	Prostatitis	.635	.403	.012	.375

CD = Crohn disease, IVW = inverse-variance weighted, MR = Mendelian randomization, PRESSO = Pleiotropy RESidual Sum and Outlier, UC = ulcerative colitis.

## 4. Discussion

This study employed a large-scale 2-sample MR analysis to systematically evaluate the causal relationships between IBD and genitourinary system diseases. Our findings indicate that CD is associated with increased risks of hypertensive renal disease, diabetic nephropathy, CKD, chronic renal failure, calculus of kidney and ureter, neuromuscular dysfunction of bladder, and hydronephrosis. Meanwhile, UC shows potential associations with a higher risk of neuromuscular dysfunction of bladder and prostatitis. These results corroborate previous observational studies and suggest causal relationships between IBD and certain genitourinary system diseases.

An analysis was performed on 13,339,065 people to determine the prevalence of urolithiasis in patients with IBD, around 6.3%. The prevalence of nephrolithiasis in CD patients is higher than that in UC and undetermined IBD patients. Calcium oxalate and uric acid are the primary components of the stones, with a smaller portion consisting of calcium phosphate stones.^[21]^ This could be because CD commonly impacts the end part of the small intestine, causing a decrease in bile acid absorption and a rise in the transportation of fatty acids to the large intestine. Fatty acids competitively bind calcium with oxalate within the colon, ultimately excreting free oxalate into the urine and subsequent stone formation.^[22]^ Furthermore, within the human colon resides a microbiome, where *Oxalobacter formigenes* are crucial for oxalate transportation and secretion. The increased permeability of colonic epithelial cells to oxalate in IBD patients diminishes the colonization capacity of *O formigenes* in the intestine, thereby creating a conducive environment for the formation of oxalate stones.^[23]^ This could increase urinary oxalate levels in IBD patients, thereby increasing the likelihood of calcium oxalate stone formation.^[24,25]^ Research by Menghan Liu et al recently demonstrated that microbial oxalate degradation pathways are downregulated in the microbiota of IBD patients, which may disrupt oxalate homeostasis in the body.^[26]^ Furthermore, research has indicated that factors like having surgery (such as colectomy), extended periods of immobility, and prolonged use of sulfasalazine can also elevate the likelihood of stone development in individuals with IBD.^[27]^ Recent research has shown a significant link between IBD and lower urinary tract symptoms, indicating that inflammation in the intestines can affect nearby pelvic organs. This relationship emphasizes the need for healthcare providers to evaluate urinary health in patients with IBD.^[28]^ Moreover, there is evidence that IBD is associated with sexual dysfunction in both men and women, impacting overall quality of life. These findings underscore the importance of considering both gastrointestinal and genitourinary symptoms in the management of IBD patients.^[29]^

Different histological forms of CKD, including immunoglobin (Ig)A nephropathy, IgM nephropathy, membranous nephropathy, focal segmental glomerulosclerosis, and anti-glomerular basement membrane glomerulonephritis, have been reported in patients with IBD and renal involvement.^[22,30]^ IgA nephropathy is a significant contributor to CKD in individuals with IBD. Pathogenesis may involve a combination of mucosal inflammation, antigenic rejection, immune stimulation, and other factors closely linked to environmental factors.^[31]^ Additional investigation and study are necessary to clarify the mechanisms behind different histological forms of CKD related to IBD. Our study preliminarily indicates a causal relationship between CD and renal failure, which differs from the nephrotoxicity induced by 5-aminosalicylic acid reported in other studies on IBD, possibly due to CD-related chronic glomerulonephritis, tubulointerstitial nephritis, and renal amyloidosis.^[32]^

For the increased risk of hypertensive kidney disease in CD, due to limited previous studies on the association between the 2, we speculate that this result may be attributed to the disruption of the intestinal mucosal barrier in IBD, leading to the induction of pro-inflammatory cytokine responses, endothelial dysfunction, and long-term vascular damage caused by the chronic inflammatory process in IBD patients, which may lead to prolonged hypertension and ultimately result in structural and functional damage to the kidneys.^[33]^

In addition, CD has been repeatedly associated with diabetes, especially type 2 diabetes, which may indirectly promote diabetic kidney disease.^[34]^ Some studies suggest that the gut-kidney axis plays a significant role between the 2.^[35]^ Additionally, specific research indicates that the kallikrein-kinin system plays a role in microvascular issues in individuals with diabetes, including the onset of diabetic nephropathy.^[36]^ Due to the chronic inflammation in the intestines associated with CD, we believe the kallikrein-kinin system is a critical factor in the connection between CD and diabetic nephropathy.

The association between CD, UC, and neuromuscular dysfunction of the bladder has yet to be discussed. Chunmei Xia et al showed that an inflammation-induced increase in the phospholipase C gamma pathway following brain-derived neurotrophic factor in bladder sensory neurons in the dorsal root ganglia is crucial in stimulating these neurons, resulting in bladder overactivity, indicating a link between IBD and bladder neuromuscular function.^[37]^ However, we did not find evidence to explain how CD contributes to neuromuscular dysfunction of bladder and further research is needed.

The mechanisms underlying prostatitis remain unclear, with literature suggesting various sources contributing to its onset, including bacterial infection, viruses, sexually transmitted pathogens, hormones, autoimmune reactions, and urinary reflux. The presence of one or more of these stimulating factors can contribute to the onset and progression of prostatitis.^[38]^ Research by Weronika Ratajczak et al discovered that intestinal flora could indirectly inhibit or promote prostate inflammation, thus impacting inflammatory marker levels.^[39]^ Additionally, individuals suffering from IBD experience ongoing inflammation, which results in an imbalance of the gut microbiota. Therefore, the interaction of the gut-prostate axis may explain the connection between the 2 conditions. And the gut-prostate axis can explain the association between gut microbiota alterations, increased intestinal epithelial permeability, and prostate diseases.^[40]^ In summary, characterized by inflammation, UC, and prostatitis exhibit various complex connections, but the precise mechanisms involved require further elucidation.

Our MR study has several limitations. Firstly, our findings only reveal causal relationships between diseases stemming from statistical analyses, thus necessitating extensive fundamental research and clinical validation. Moreover, detailed investigations into the mechanisms underlying these results are crucial. Secondly, our study was restricted to individuals of European ancestry, which could impact the applicability of the findings to different populations. Finally, despite the rigorous measures taken to detect outlier variants and prevent pleiotropy, the influence of pleiotropy cannot be eradicated, possibly because of numerous genetic variants’ intricate and ambiguous biological roles.

## 5. Conclusion

In conclusion, our study suggests a casual effects between IBD and GDs through MR analysis, which needs further investigation to prove.

## Author contributions

**Conceptualization:** Song Wang, Hongliang Cao.

**Formal analysis:** Song Wang, Hongliang Cao, Chengdong Shi.

**Funding acquisition:** Song Wang.

**Investigation:** Song Wang, Hongliang Cao, Chengdong Shi.

**Methodology:** Song Wang, Hongliang Cao, Gang Yang.

**Resources:** Song Wang, Hongliang Cao, Chengdong Shi.

**Software:** Song Wang, Hongliang Cao, Chengdong Shi, Xianyu Dai, Gang Yang.

**Supervision:** Song Wang, Chengdong Shi.

**Validation:** Song Wang, Hongliang Cao, Chengdong Shi, Xianyu Dai, Bo Yuan.

**Visualization:** Song Wang, Hongliang Cao, Chengdong Shi, Xianyu Dai, Bo Yuan, Gang Yang.

**Writing – original draft:** Song Wang, Hongliang Cao, Chengdong Shi, Xianyu Dai, Bo Yuan, Gang Yang.

**Writing – review & editing:** Song Wang, Hongliang Cao, Chengdong Shi, Xianyu Dai, Bo Yuan, Gang Yang.




































