# Supplementary material for: Mendelian randomization analyses explore the relationship between inflammatory bowel disease and genitourinary diseases
Source: Medicine (Baltimore). 2026 May 15;105(20):e48759. doi: 10.1097/MD.0000000000048759 (PMC13183042; doi:10.1097/MD.0000000000048759)
Supplement: Supplementary file 6 [file medi-105-e48759-s006.docx]

**
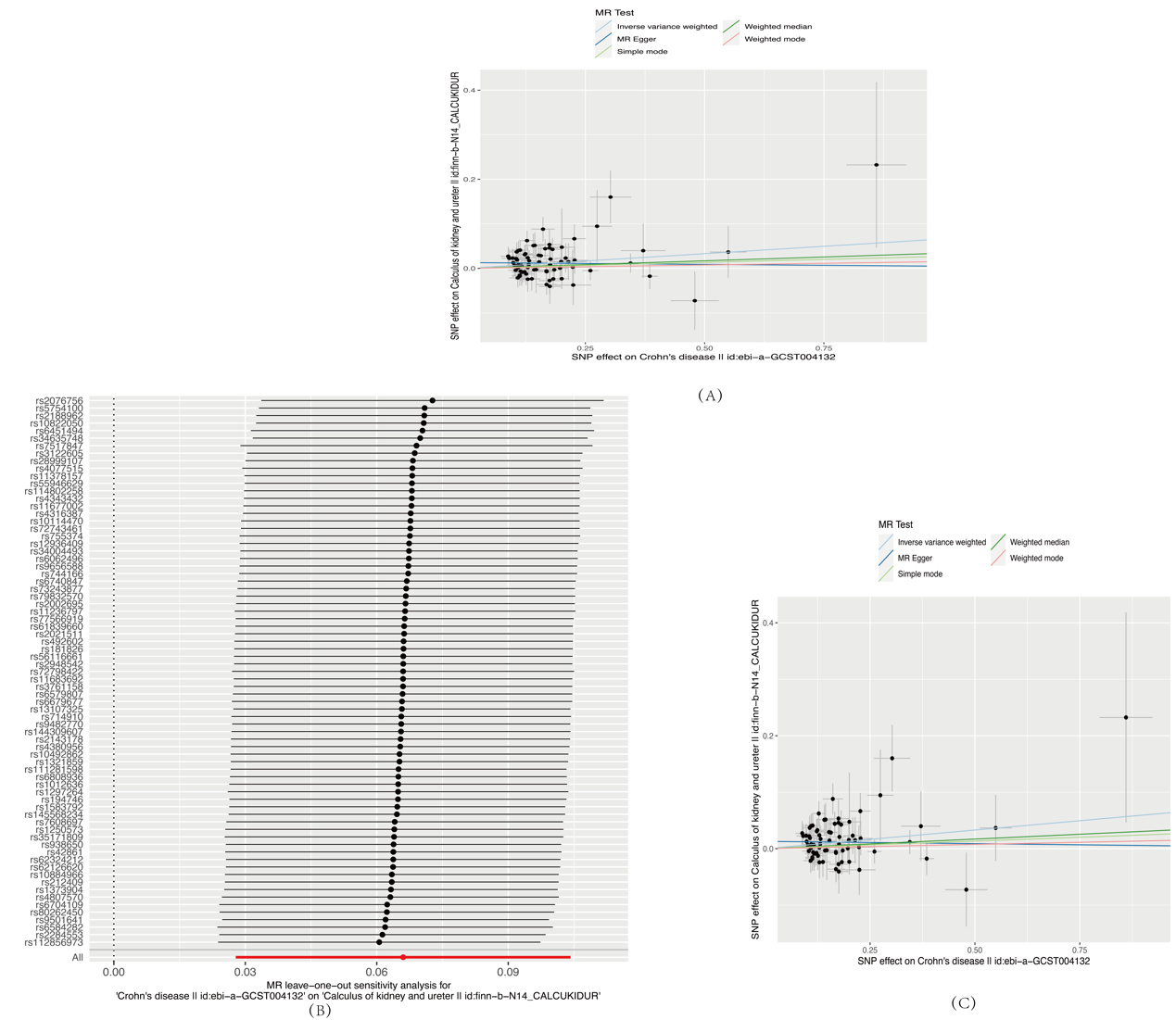
**

**Supplementary Figure 5**: (A) Scatter plot between Crohn's disease and calculus of kidney and ureter; (B) Leave-one-out plot between Crohn's disease and calculus of kidney and ureter; (C) Funnel plot between Crohn's disease and calculus of kidney and ureter.
